# A Commentary on “Associations of the NPAR index with breast cancer incidence and mortality (NHANES 2001–2018)”

**DOI:** 10.1097/JS9.0000000000003563

**Published:** 2025-10-13

**Authors:** Guo-Ming Zhang

**Affiliations:** Department of Laboratory Medicine, Shuyang Hospital, The Affiliated Shuyang Hospital of Xuzhou Medical University, Shuyang, Jiangsu, People’s Republic of China

*Dear Editor*,

This letter complies with the TITAN Guidelines 2025 on declaration and responsible use of AI[1]. I read with great interest the recent article by Su *et al* that explored the neutrophil-percentage-to-albumin ratio (NPAR) as a predictor of breast cancer (BC) incidence and mortality via NHANES data from 2001 to 2018^[2]^. The authors should be commended for addressing an important clinical question and applying machine-learning methods to a large national dataset. However, several methodological and interpretive issues require attention before the NPAR can be considered a reliable biomarker for clinical use.

First, there is potential confounding by hypoalbuminemia. Albumin (ALB) is a nonspecific marker that decreases for a variety of reasons. Malnutrition, chronic liver disease, and protein-losing renal disorders all lower the serum ALB concentration and thereby elevate the NPAR independently of tumor biology[3]. The NHANES contains variables such as the estimated glomerular filtration rate (eGFR), urinary ALB-to-creatinine ratio, body mass index (BMI), and liver disease history. These data could be used to adjust for or exclude participants with major non-neoplastic causes of hypoalbuminemia. At a minimum, sensitivity analyses excluding individuals with an ALB concentration <3.5 g/dL, an eGFR <60 mL/min/1.73 m^2^, or self-reported liver disease are necessary. Without this information, it remains unclear whether the observed associations reflect tumor-related biology or background comorbidities.

Second, associations should not be misinterpreted as causation. The manuscript occasionally uses causal phrasing such as “elevated NPAR increases breast cancer odds.” Given the cross-sectional nature of NHANES, such language is inappropriate. The data can demonstrate associations but cannot establish temporal sequences or causalities. Reverse causation – where undiagnosed or early BC leads to systemic inflammation, weight loss, or ALB decline – remains plausible. Conclusions should be restated as “NPAR is associated with” rather than implying mechanistic or causal effects[4].

The third factor is the impact of acute infection and inflammatory states. Acute infections and systemic inflammation can transiently alter neutrophil counts, causing either neutrophilia or, in severe cases, neutropenia. They can also lower ALB levels through inflammatory pathways^[5,6]^. Such perturbations may substantially affect the NPAR independent of cancer risk. Excluding individuals with leukocytosis, neutropenia, or elevated inflammatory markers (e.g., (C-reactive protein) CRP >10 mg/L) would strengthen confidence that the NPAR reflects long-term cancer-associated processes rather than acute illness. Sensitivity analyses removing participants with extreme neutrophil values should also be presented.

Fourth, deficiencies in receiver operating characteristic (ROC) analysis and reporting exist. The reported discrimination is striking, with area under the ROC curve (AUCs) approaching 0.995, but the absence of 95% confidence intervals (CIs) undermines interpretation. International standards recommend that ROC analyses include CIs, cut-off selection methodology, and formal statistical comparisons between curves^[7–9]^. The authors should:

1. AUC values with 95% CIs were reported via nonparametric (DeLong) or bootstrap methods.

2. ROC curves are compared formally via DeLong’s test for correlated samples[7].

3. The sensitivity, specificity, and predictive values at the optimal cut-off are presented, ideally on the basis of Youden’s index[8], with CIs obtained by bootstrapping[9].

Fifth, the machine learning models were validated and calibrated. The extremely high AUCs raise concerns about optimism bias. While synthetic minority oversampling (SMOTE) may balance classes, it can also inflate performance estimates if applied before cross-validation. Authors should present results on the original, unaltered test set alongside SMOTE-adjusted analyses. Calibration metrics (calibration slope/intercept, Brier score), calibration plots, and decision-curve analysis are needed to assess whether predictions align with observed outcomes and offer real clinical benefit[10]. Transparent adherence to the TRIPOD guidelines for prediction model reporting would further enhance credibility[4].

In summary, while the NPAR is an appealing and inexpensive biomarker, its interpretation is easily confounded by malnutrition, liver dysfunction, renal impairment, and acute inflammatory states. The study would be strengthened by excluding or adjusting for these conditions, avoiding causal language, reporting ROC analyses with CIs and statistical comparisons, and providing calibration and decision curve assessments with performance tested on unaltered data. Addressing these issues will improve the methodological rigor and enhance the clinical utility of the findings.

## Data Availability

Not applicable.
